# Streptozotocin induces renal proximal tubular injury through p53 signaling activation

**DOI:** 10.1038/s41598-023-35850-w

**Published:** 2023-05-29

**Authors:** Kunihiro Nakai, Minato Umehara, Atsushi Minamida, Hiroko Yamauchi-Sawada, Yasuto Sunahara, Yayoi Matoba, Natsuko Okuno-Ozeki, Itaru Nakamura, Tomohiro Nakata, Aya Yagi-Tomita, Noriko Uehara-Watanabe, Tomoharu Ida, Noriyuki Yamashita, Michitsugu Kamezaki, Yuhei Kirita, Eiichi Konishi, Hiroaki Yasuda, Satoaki Matoba, Keiichi Tamagaki, Tetsuro Kusaba

**Affiliations:** 1 Department of Nephrology, Graduate School of Medical Science, Kyoto Prefectural University of Medicine, 465 Kajii-cho, Kamigyo-ku, Kyoto, 602-8566 Japan; 2 Department of Surgical Pathology, Graduate School of Medical Science, Kyoto Prefectural University of Medicine, Kyoto, Japan; 3 Department of Gastroenterology and Hepatology, Graduate School of Medical Science, Kyoto Prefectural University of Medicine, Kyoto, Japan; 4 Department of Cardiovascular Medicine, Graduate School of Medical Science, Kyoto Prefectural University of Medicine, Kyoto, Japan

**Keywords:** Cell signalling, Mechanisms of disease, DNA damage and repair, Acute kidney injury, Toxin-induced nephropathy, Chemotherapy

## Abstract

Streptozotocin (STZ), an anti-cancer drug that is primarily used to treat neuroendocrine tumors (NETs) in clinical settings, is incorporated into pancreatic β-cells or proximal tubular epithelial cells through the glucose transporter, GLUT2. However, its cytotoxic effects on kidney cells have been underestimated and the underlying mechanisms remain unclear. We herein demonstrated that DNA damage and subsequent p53 signaling were responsible for the development of STZ-induced tubular epithelial injury. We detected tubular epithelial DNA damage in NET patients treated with STZ. Unbiased transcriptomics of STZ-treated tubular epithelial cells in vitro showed the activation of the p53 signaling pathway. STZ induced DNA damage and activated p53 signaling in vivo in a dose-dependent manner, resulting in reduced membrane transporters. The pharmacological inhibition of p53 and sodium-glucose transporter 2 (SGLT2) mitigated STZ-induced epithelial injury. However, the cytotoxic effects of STZ on pancreatic β-cells were preserved in SGLT2 inhibitor-treated mice. The present results demonstrate the proximal tubular-specific cytotoxicity of STZ and the underlying mechanisms in vivo. Since the cytotoxic effects of STZ against β-cells were not impaired by dapagliflozin, pretreatment with an SGLT2 inhibitor has potential as a preventative remedy for kidney injury in NET patients treated with STZ.

## Introduction

Streptozocin/Streptozotocin (STZ), a glucose analog, is classified as an anti-cancer drug of alkylating agents and is one of the major classes of cytotoxic drugs that are primarily used to treat neuroendocrine tumors (NETs)^1,2^. STZ is incorporated into pancreatic β-cells through glucose transporter type 2 (GLUT2), thereby promoting DNA damage, reactive oxygen species (ROS) production, mitochondrial dysfunction, and subsequent apoptosis^3–5^. Based on the findings of clinical trials, the combination of STZ and 5-fluorouracil is commonly used to treat patients with well-differentiated NETs^6,7^.

Due to the high expression of GLUT2 in the liver and kidneys, common side effects of STZ occur in these organs^2,8^. Besides renal dysfunction, hypophosphatemia and renal glycosuria sometimes develop in patients treated with STZ^9^. Non-diabetic glycosuria or hypophosphatemia due to hyperphosphaturia is caused by decreases in sodium-glucose transporters or sodium-phosphate co-transporters, respectively, which are predominantly expressed at the apical membrane of proximal tubular epithelial cells. Since the loss of or a decrease in brush border membrane transporters is an initial response of acute kidney injury^10–13^, glycosuria or hypophosphatemia after STZ treatment may reflect the latent proximal tubular injury, which is underestimated or ignored in clinical settings.

In experimental fields, STZ is commonly used as a diabetogenic agent to destroy pancreatic β-cells, resulting in the development of type 1 diabetes^14,15^. However, in analyses of renal phenotypes in STZ-induced type 1 diabetic mice, one potential concern is renal toxicity induced by the administration of STZ^14–16^. Another concern is that susceptibility to STZ varies among rodent types and genetic backgrounds^15^. Therefore, optimizing the dose or schedule of the administration of STZ may prevent its nephrotoxicity^15^; however, difficulties are associated with establishing whether the renal phenotypes in STZ-induced type 1 diabetic mice are attributed to high blood glucose or direct cytotoxicity by STZ^16^.

In consideration of the increased usage of STZ to treat NETs, a more detailed understanding of the underlying mechanisms of renal toxicity by STZ is essential for preventing its adverse effects. Although experimental findings on STZ-induced nephrotoxicity have already been reported^16–19^, the underlying molecular mechanisms remain unclear. In the present study, we performed unbiased transcriptomics on STZ-treated tubular epithelial cells in vitro as well as in in vivo experiments, with a focus on DNA damage and subsequent p53 signaling.

## Results

### DNA damage to tubular epithelial cells in NET patients treated with STZ

**Figure 1:**
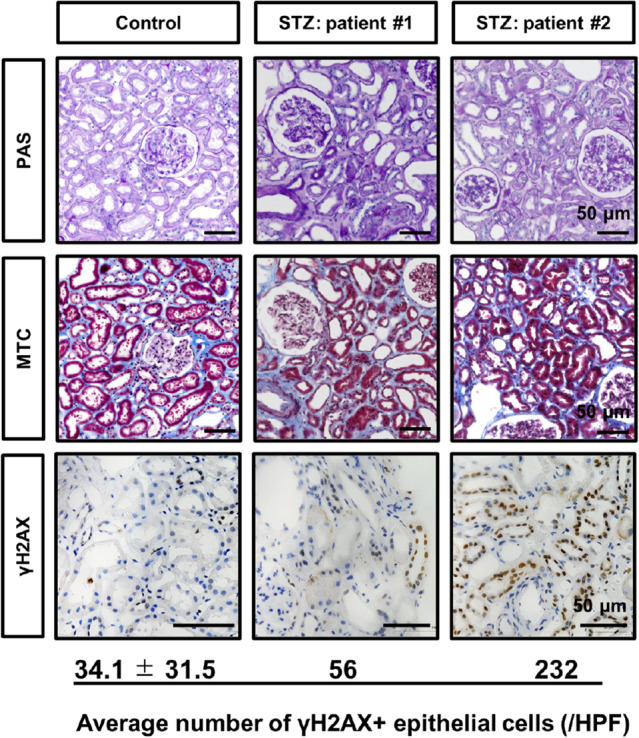
DNA damage in tubular epithelial cells in neuroendocrine tumor (NET) patients treated with STZ. Microscopic images of human kidney biopsy samples subjected to immunostaining for γH2AX as well as PAS and Masson trichrome staining. Number of γH2AX + tubular epithelia was counted high magnification pictures of three thin basement membrane disease patients as healthy control and two STZ-treated NET patients (#1 and #2). Data is means ± SD. Bar = 50 μm.

We initially examined the renal phenotypes of eight NET patients treated with STZ in our institution. Average age and serum creatine levels were 62.0 ± 10.9 years and 0.87 ± 0.12 mg/dl, respectively (Supplementary Table 1). One patient developed diabetes after STZ treatment due to surgical resection of pancreas tumor. In comparisons with blood glucose levels at the time of urine collection, 7 out of 8 patients presented with inadequately positive urinary glucose. Five out of 7 patients and 6 out of 7 patients presented low serum phosphorus and low uric acid levels, respectively (Supplementary Table 1). Renal histology was evaluated in 2 patients. PAS and Masson trichrome staining showed moderate interstitial fibrosis and tubular injury characterized by the loss of the brush border and flattened tubular epithelial cells (Fig. 1). Since DNA damage is one of the major pathophysiologies among the cytotoxic effects induced by STZ, we performed immunostaining for γH2AX, a marker of DNA damage. γH2AX staining revealed positive nuclear staining in tubular epithelial cells in patients, whereas not in the uninjured control (Fig. 1).

### p53 up-regulation in STZ-treated tubular epithelial injury in vitro

**Figure 2:**
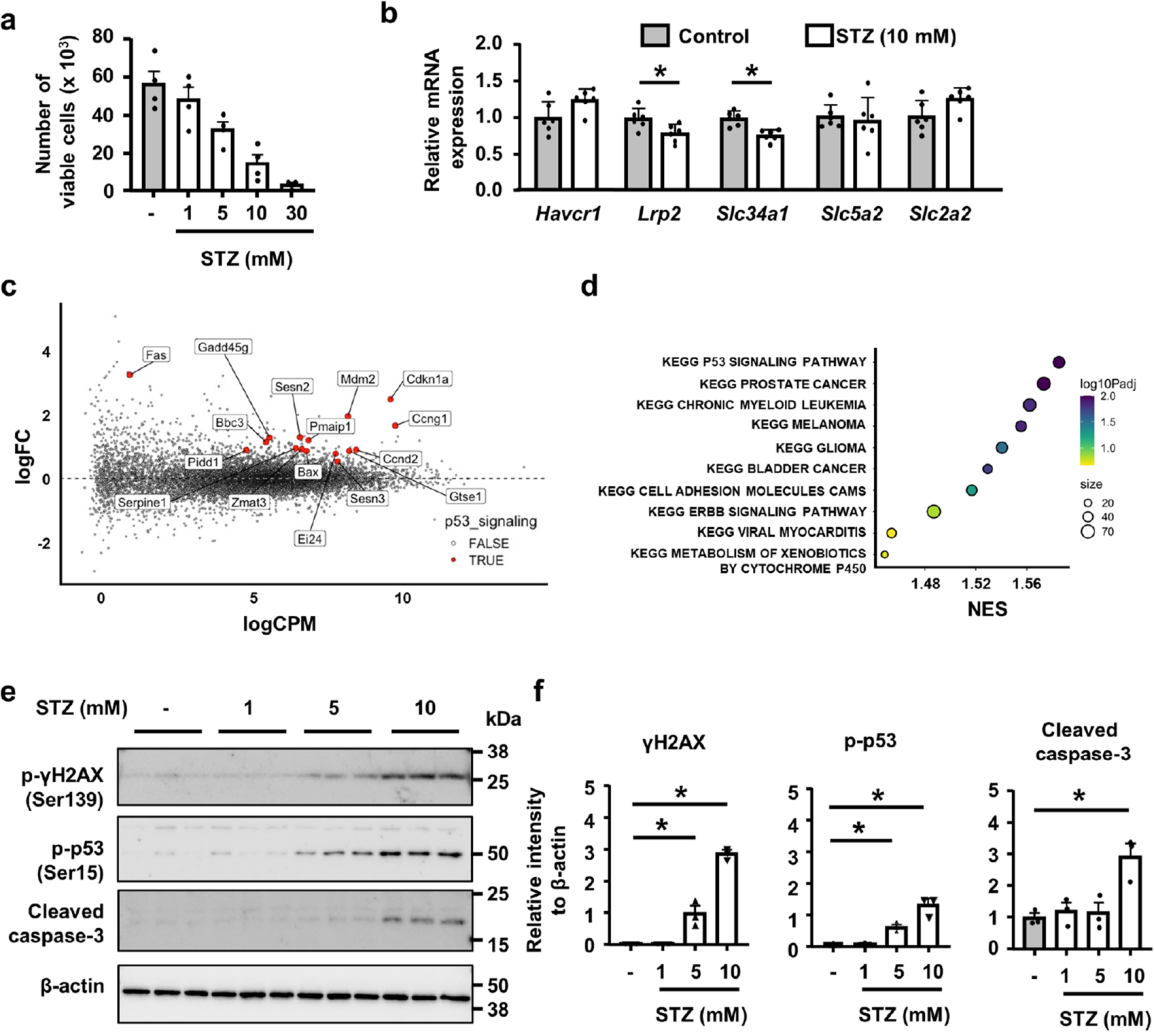
Up-regulation of p53 signaling in STZ-treated tubular epithelial cells. (**a**) The number of viable NRK-52E cells was reduced 24 h after the STZ treatment in a dose-dependent manner. (**b**) qPCR of RNA for the representative markers of injured and healthy tubules. (**c**) The RNA sequence of STZ-treated NRK-52E cells. Highlighted genes are significantly differentially expressed genes included in the p53 signaling pathway. (**d**) Enrichment analysis of the KEGG pathways for differentially expressed genes between untreated and STZ-treated cells. The top 10 significantly enriched KEGG pathways are presented. (**e**) Western blot of protein lysates from NRK-52E cells for γH2AX, phospho-p53, cleaved caspase-3, and GAPDH. Representative images of n = 3. (**f**) The optical densities of γH2AX, phosphor-p53, and cleaved caspase-3 bands were normalized against that of GAPDH. In all groups, data are means ± SEM. Statistical analyses were performed using an unpaired *t*-test in (**b**) and Dunnett's post hoc test was used for multiple comparisons in (**f**). * *p* < 0.05.

We examined the cytotoxic response to STZ in NRK52E cells, and found that STZ reduced cell viability in a dose-dependent manner (Fig. 2a). qPCR revealed that STZ did not increase the expression of the proximal tubular injury marker *Havcr1* (encoding Kim-1), but slightly reduced that of some mature tubular epithelial markers *Slc34a1*, and *Lrp2* (encoding NaPi2a and megalin, respectively) (Fig. 2b). To identify the responsible cell signaling for cytotoxicity of STZ, we then investigated differences in the transcriptome between NRK52E cells treated with 10 mM of STZ or vehicle for 24 h by RNA sequence. An unsupervised analysis of gene expression revealed 266 out of 18,930 genes that exhibited differential expression between two groups (false detection rate-corrected *p*-value < 0.05, absolute fold change > 2) (Fig. 2c).

We annotated gene expression using the Kyoto Encyclopedia of Genes and Genomes (KEGG) pathway, and identified 12 significantly enriched pathways (threshold *p*-value < 0.05) (Fig. 2d). The p53 signaling pathway was the most highly up-regulated in STZ-treated cells (Fig. 2d, Supplementary Fig. 1). Western blotting of cell lysates revealed that STZ up-regulated γH2AX expression and p53 phosphorylation in a dose-dependent manner (Fig. 2e,f).

### Dose-dependent tubular injury by STZ in vivo

**Figure 3:**
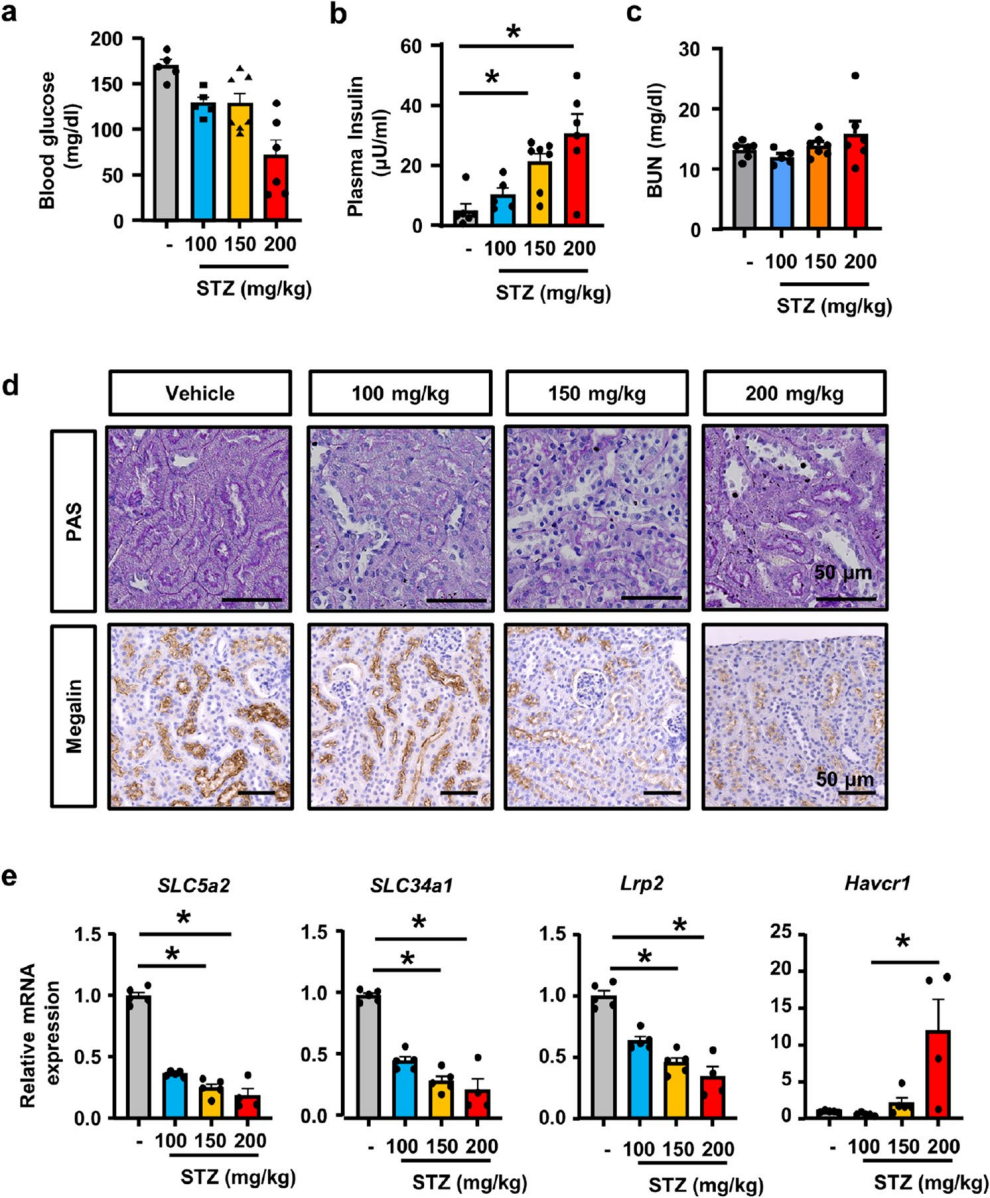
Dose-dependent tubular injury by STZ in vivo*.* (**a**) Decreases in blood glucose levels in mice treated with 200 mg/kg of STZ. (**b**) Plasma insulin levels were higher in high dose of STZ-treatment groups. (**c**) BUN levels did not differ among experimental groups. (**d**) Microscopic images of kidney tissue with PAS staining and immunostaining for megalin showed increases in the tubules with the loss of the brush border and decreases in megalin expression in STZ-treated mice. (**e**) qPCR of RNA from kidney tissue for the representative markers of injured and healthy tubules (apical membrane sodium transporters). In all groups, data are means ± SEM. Statistical analyses were performed using Dunnett's post hoc test was used for multiple comparisons. N = 5 to 7 mice per group. * *p* < 0.05, Bar = 50 μm in (**c**).

To investigate whether STZ induces tubular injury in vivo, we injected various concentrations of STZ into mice. Twenty-four hours after the STZ injection, blood glucose levels decreased (Fig. 3a) and plasma insulin levels increased in mice treated with the higher dose of STZ (Fig. 3b), reflecting insulin release as a consequence of pancreatic β-cell death. Although serum BUN did not differ among experimental groups (Fig. 3c), the kidneys of mice that received a single injection of STZ exhibited tubular injury that was characterized by the loss of the brush border, which was more severe in mice treated with a higher dose of STZ (Fig. 3d). qPCR of whole kidney RNA revealed the decreased expression of the mature tubular epithelial markers *Slc5a2* (encoding SGLT2), and the increased expression of *Havcr1* in STZ-treated mice (Fig. 3e). We also confirmed a reduction in immunostaining for megalin in STZ-treated mice (Fig. 3d).

### Dose-dependent DNA damage by STZ in tubular epithelial cells

**Figure 4:**
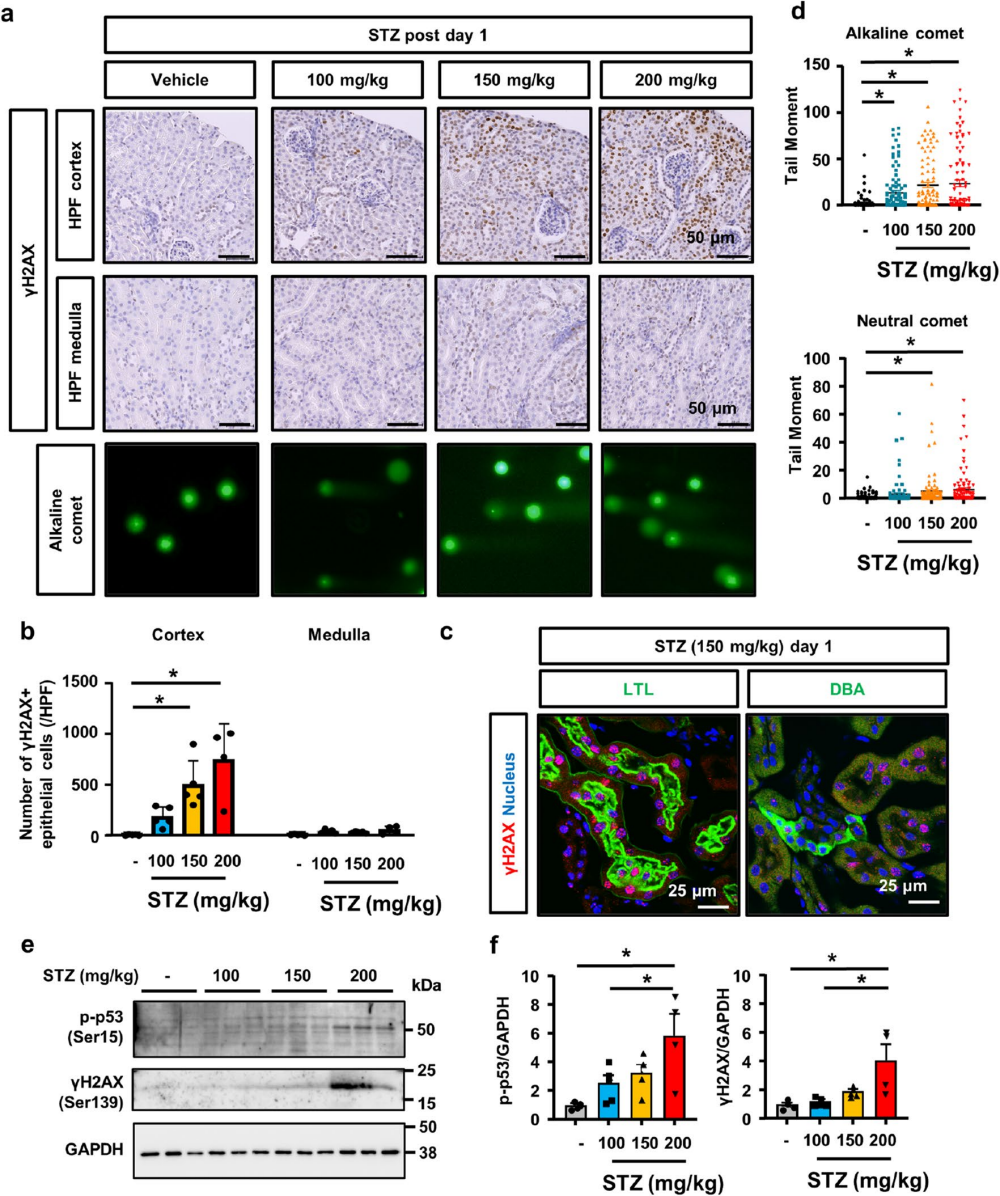
Dose-dependent DNA damage by STZ in vivo*.* (**a**) Microscopic images of kidneys immunostained for γH2AX and representative images of the comet assay of kidney cells isolated from mice 24 h after the STZ treatment. (**b**) Separate quantification of nuclear γH2AX-positive cells in the kidney cortex and medulla. (**c**) Immunofluorescence pictures of γH2AX and proximal tubular epithelial marker (LTL) or collecting duct marker (DBA) in STZ-treated mice. (**d**) A quantitative analysis (alkaline comet: n = 100 each, neutral comet: n = 50 each). (**e**) Western blot of kidney tissue lysates for γH2AX, phospho-p53, and GAPDH. Representative images of n = 3. (f) The optical densities of γH2AX and phospho-p53 bands were normalized against that of GAPDH. N = 5 to 7 mice per group. In all groups, data are means ± SEM. Statistical analyses were performed using Dunnett's post hoc test was used for multiple comparisons. * *p* < 0.05, Bar = 50 μm in (a) and 25 μm in (**c**).

We examined tubular epithelial DNA damage and subsequent p53 activation in vivo. Immunostaining for γH2AX revealed positive nuclear staining in the kidney tissue of mice treated with the higher dose of STZ (Fig. 4a). The localization of positive staining was limited to the renal cortex, not the renal medulla (Fig. 4a,b, Supplementary Fig. 2). Immunofluorescence staining with Lotus Tetragonolobus Lectin (LTL), a proximal tubule marker, showed that most of the γH2AX positive cells co-stained with LTL, while immunofluorescence for Dolichos Biflorus Agglutinin (DBA), a collecting duct marker, showed that γH2AX positive cells rarely co-stained with DBA (Fig. 4c). These indicates STZ-induced DNA damage predominantly occurs in the cortical proximal tubules. To confirm STZ-induced DNA damage and elucidate the type of DNA damage in the kidneys, we performed a comet assay of whole kidney tissue. The comet tail moment under alkaline conditions (an alkaline comet) reflects DNA double-stranded breaks (DSB) and single-stranded breaks (SSB), whereas that under neutral conditions (a neutral comet) only reflects DNA DSB^20^. Alkaline comet tail moments increased in kidney cells from STZ-treated mice in a dose-dependent manner (Fig. 4a,d). Neutral comet tail moments also increased in the kidney cells of STZ-treated mice in a dose-dependent manner; however, the difference among these groups was less for neutral comet tail moments (Fig. 4d). This result suggests that DNA DSB and SSB occurred in the kidneys after the administration of STZ in a dose-dependent manner. We also confirmed the up-regulation of γH2AX expression and p53 phosphorylation in whole kidney lysates from STZ-treated mice in a dose-dependent manner (Fig. 4e,f).

### Blood glucose-independent tubular epithelial injury by STZ

**Figure 5:**
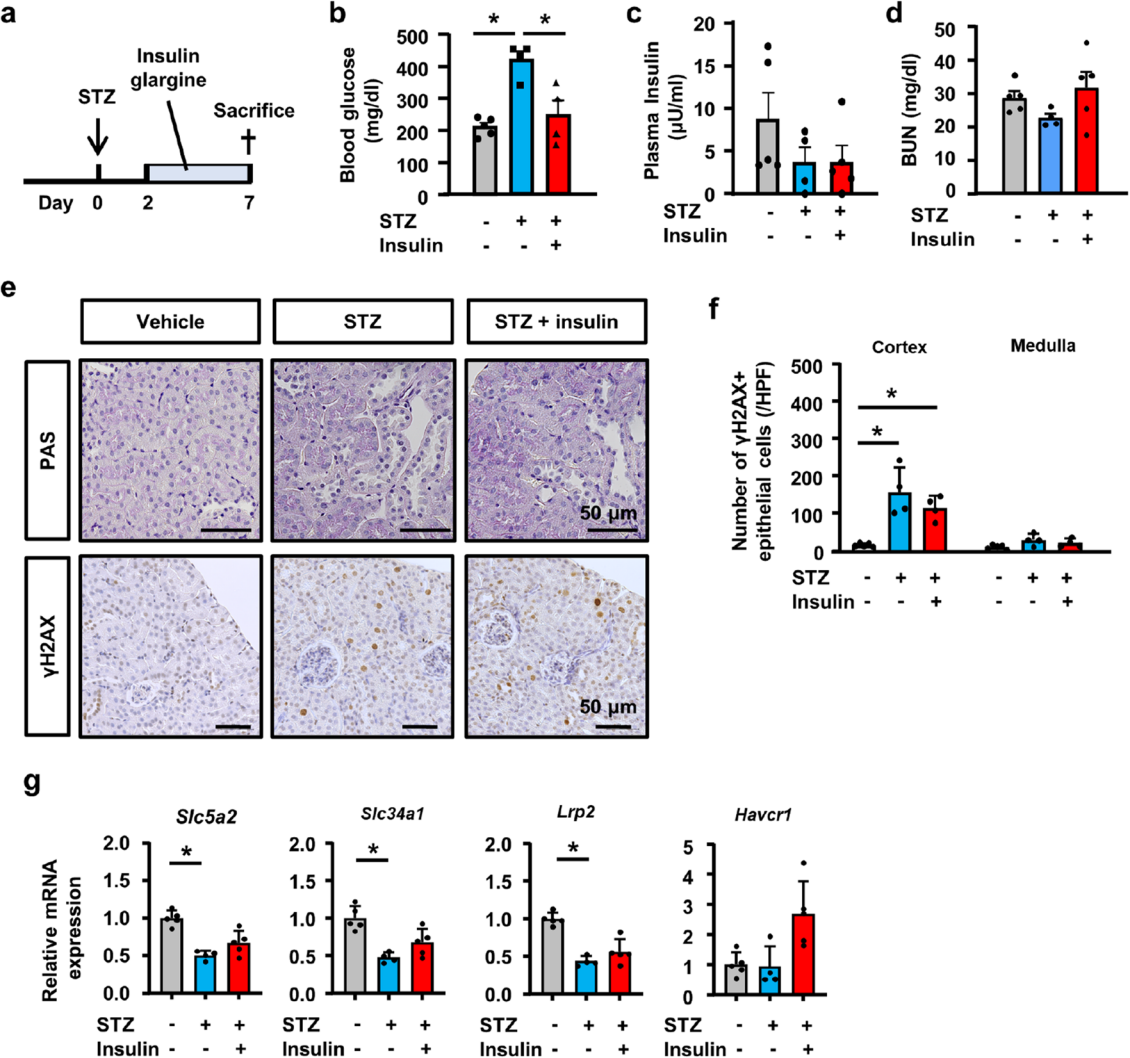
Blood glucose independent of tubular injury by STZ. (**a**) Experimental scheme. Two days after the STZ (150 mg/kg) treatment, insulin glargine was administered at approximately 200 mg/dl to control blood glucose levels, and mice were sacrificed on day 7. (**b**) Blood glucose levels increased in mice treated with STZ, but were decreased by the administration of insulin glargine. (**c**,**d**) Plasma insulin levels (**c**) and BUN levels (**d**) did not differ among experimental groups. (**e**) Microscopic images of kidney tissue stained with PAS and immunostained for γH2AX showed increases in tubules with the loss of the brush border and nuclear γH2AX-positive cells in STZ-treated mice with or without insulin glargine. (**f**) Separate quantification of nuclear γH2AX-positive cells in the kidney cortex and medulla. (**g**) qPCR of RNA from kidney tissue for the representative markers of injured and healthy tubules. In all groups, data are means ± SEM. Statistical analyses were performed using Dunnett's post hoc test was used for multiple comparisons. N = 4 to 5 mice per group. * *p* < 0.05, Bar = 50 μm in (**e**).

To evaluate STZ-induced tubular injury in the subacute phase in vivo, we extended the observation period to 7 days after the STZ injection (Fig. 5a). To distinguish high blood glucose-induced tubular injury, we administered insulin glargine to reduce blood glucose levels in STZ-injected mice (Fig. 5a,b). Plasma insulin levels slightly decreased in STZ-treated mice, but there was not statistical significance (Fig. 5c). Serum BUN did not differ among experimental groups (Fig. 5d). Seven days after the single-dose STZ injection (150 mg/kg), the kidneys of mice exhibited tubular injury with the loss of the brush border; however, no significant difference was observed between the STZ injection with or without insulin (Fig. 5e). Immunostaining for γH2AX revealed positive nuclear staining in STZ with or without insulin (Fig. 5e,f). qPCR of whole kidney RNA revealed the slightly decreased expression of mature tubular epithelial markers (*Slc5a2, Slc34a1,* and *Lrp2*), and no significant change in the expression of *Havcr1* in STZ-treated mice (Fig. 5g). This result indicates that STZ-induced tubular injury occurred independently of blood glucose levels in the subacute phase.

### Effects of the co-administration of pifithrin-α or dapagliflozin on STZ-induced tubular injury

**Figure 6:**
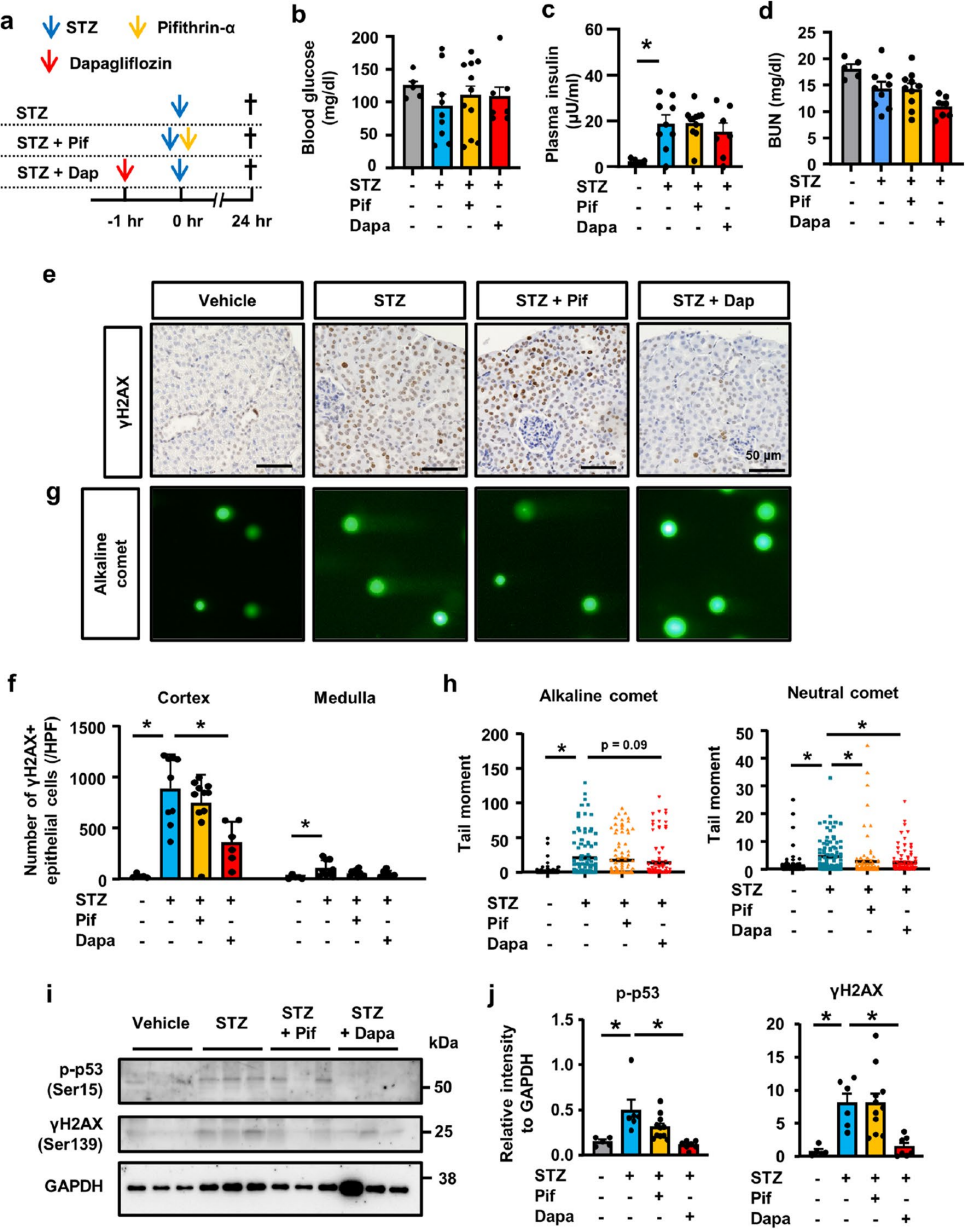
Pharmacological inhibition of p53 signaling and SGLT2 ameliorated STZ-induced DNA damage in vivo. (**a**) Experimental scheme. Pifithrin-α was administered at the same time as STZ (150 mg/kg). Dapagliflozin was administered 2 h before STZ. Mice were sacrificed 24 h after the administration of STZ. N = 5 to 11 mice per group. (**b**) Blood glucose levels were similar among all experimental groups. (**c**) Plasma insulin levels were higher in STZ-treatment groups. (**d**) BUN levels did not differ among experimental groups. (**e**) Microscopic images of kidney tissue immunostained with γH2AX. (**f**) Separate quantification of nuclear γH2AX-positive cells in the kidney cortex and medulla. (**g**) Representative images of the comet assay of kidney cells isolated from mice and (**h**) a quantitative analysis (alkaline comet: n = 100 each, neutral comet: n = 50 each). (**i**) Western blots of kidney tissue lysates for γH2AX, phospho-p53, and GAPDH. Representative images of n = 3. (**j**) The optical densities of γH2AX and phosphor-p53 bands were normalized against that of GAPDH. In all groups, data are the means ± SEM. Statistical analyses were performed using Dunnett's post hoc test was used for multiple comparisons. * *p* < 0.05, Bar = 50 μm in (**e**).

Based on the up-regulation of p53 signaling in STZ-induced tubular injury, we examined the preventative effects of the pharmacological inhibition of p53 by pifithrin-α (Fig. 6a). Previous studies demonstrated that phlorizin, a SGLT2 inhibitor, prevented STZ-induced tubular injury^19^. Therefore, we also investigated whether the pre-administration of dapagliflozin, a selective SGLT2 inhibitor, inhibited STZ-induced DNA damage and subsequent tubular injury (Fig. 6a). Blood glucose levels and BUN levels did not significantly differ among the experimental groups (Fig. 6b,d). Plasma insulin levels increased in mice treated with STZ (Fig. 6c). We also assessed DNA damage in the kidneys. Immunostaining for γH2AX revealed that STZ-induced positive nuclear staining was ameliorated by dapagliflozin, but not by pifithrin-α, (Fig. 6e,f). The comet assay showed that increases in alkaline and neutral comet tail moments were slightly attenuated by dapagliflozin (Fig. 6g,h). We also confirmed that the STZ-induced up-regulation of γH2AX expression was ameliorated by dapagliflozin, but not by pifithrin-α. In contrast, STZ-induced p53 phosphorylation was ameliorated by pifithrin-α and dapagliflozin (Fig. 6i,j).

**Figure 7:**
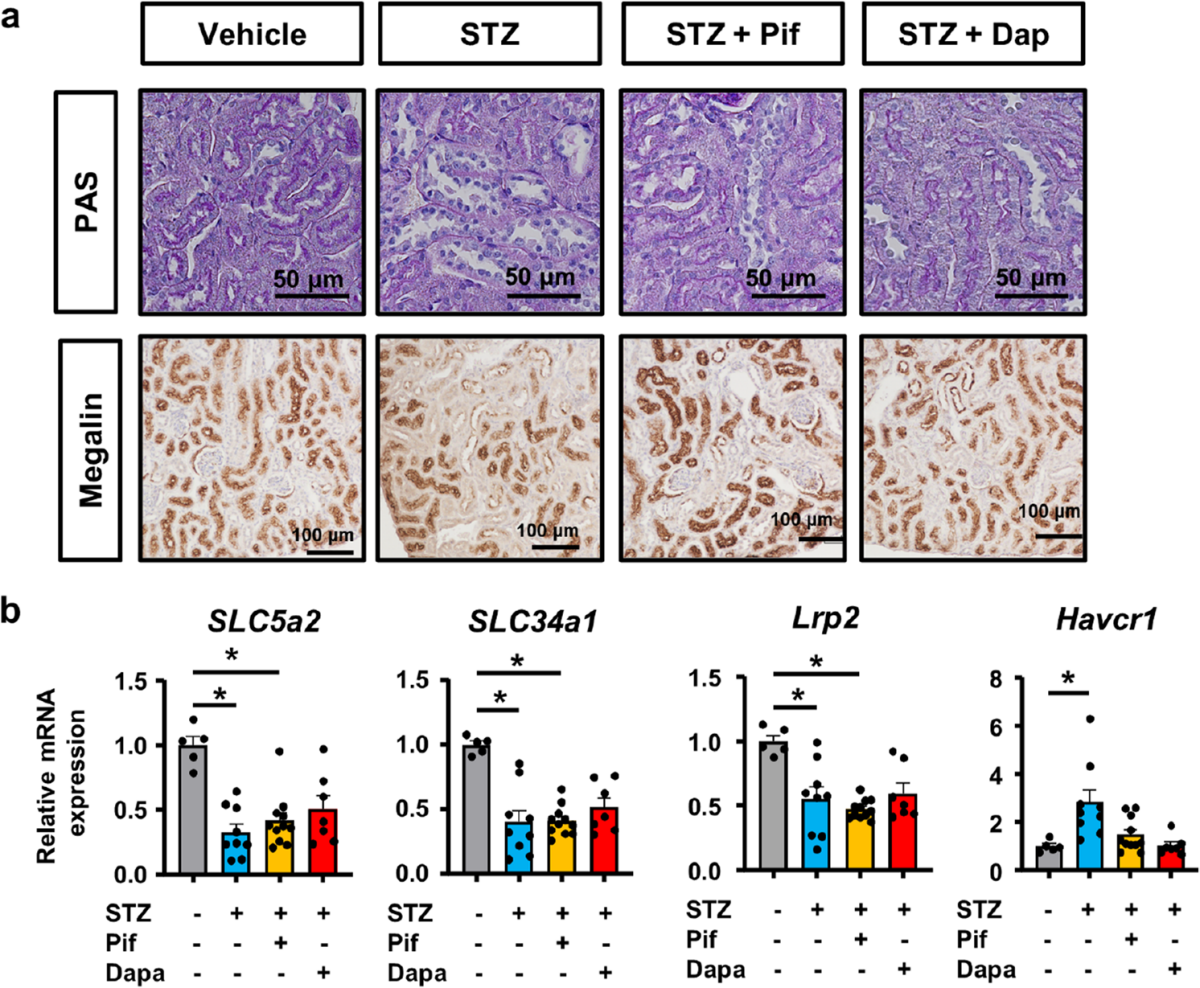
Pharmacological inhibition of p53 signaling and SGLT2 ameliorated STZ-induced tubular injury in vivo*.* (**a**) Microscopic images of kidney tissue stained with PAS and immunostained for megalin. (**b**) qPCR of RNA from kidney tissue for the representative markers of injured and healthy tubules (apical membrane sodium transporters). In all groups, data are the means ± SEM. Statistical analyses were performed using Dunnett's post hoc test was used for multiple comparisons. N = 5 to 11 mice per group. * *p* < 0.05, Bar = 50 μm in images of PAS staining and 100 μm in images of immunostaining for megalin in (**c**).

In a histological analysis, PAS staining of the kidneys demonstrated that STZ-induced tubular injury was ameliorated by pifithrin-α and dapagliflozin (Fig. 7a). Immunostaining for megalin showed that pifithrin-α and dapagliflozin preserved the expression of megalin in STZ-treated mice (Fig. 7a). qPCR revealed decreases in the expression of mature tubular epithelial markers (*Slc5a2*, *Slc34a1*, and *Lrp2)* that did not change in mice treated with pifithrin-α or dapagliflozin (Fig. 7b). However, the increased expression of *Havcr1* by STZ was slightly attenuated in mice treated with pifithrin-α or dapagliflozin (Fig. 7b).

**Figure 8:**
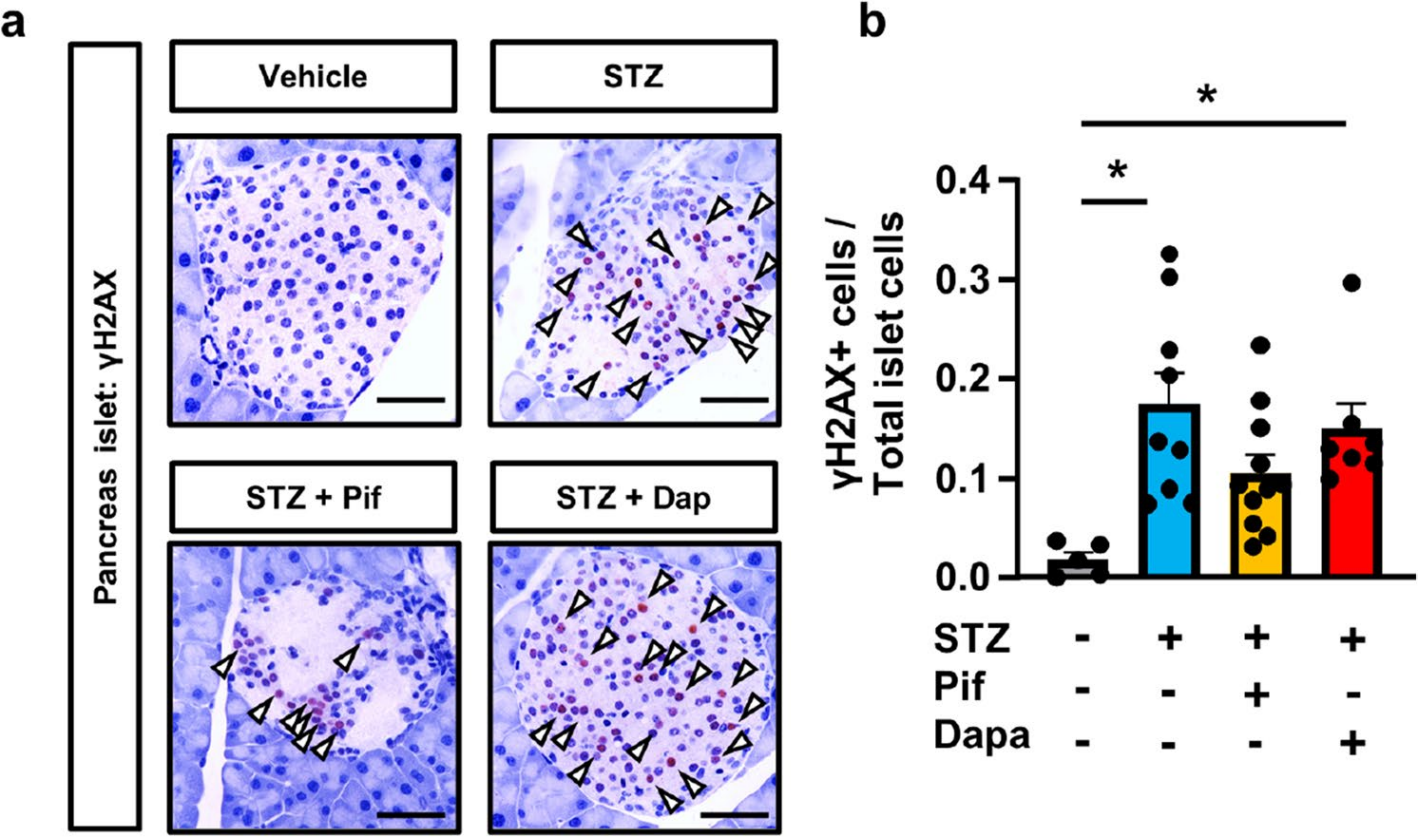
STZ induced pancreatic islet DNA damage. (**a**) Microscopic images of the pancreas immunostained for γH2AX. Arrowheads indicate γH2AX + nuclei. (**b**) Separate quantification of nuclear γH2AX-positive cells in pancreatic islets. In all groups, data are the means ± SEM. Statistical analyses were performed using Dunnett's post hoc test was used for multiple comparisons. N = 5 to 11 mice per group. * *p* < 0.05, Bar = 50 μm in (**a**).

Regarding the future clinical application of these renoprotective drugs against STZ-induced kidney injury, we evaluated the effects of the co-administration of STZ and pifithrin-α or dapagliflozin on pancreatic islet cells. Immunostaining for γH2AX revealed positive nuclear staining within the islets in all experimental groups treated with STZ (Fig. 8a). The percentage of γH2AX + cells was similar among STZ-treated groups (Fig. 8b). This result indicates that the preventative effects of dapagliflozin on DNA damage were observed in the kidneys, but not in the pancreas, which does not express SGLT2.

## Discussion

The present study investigated the mechanisms underlying STZ-induced tubular injury in vitro and in vivo. A transcriptomic analysis showed that p53 signaling was activated in STZ-treated tubular epithelial cells. STZ induced DNA damage in tubular epithelial cells in a dose-dependent manner, and its localization was limited to within the kidney cortex. The pharmacological inhibition of p53, as well as SGLT2, reduced STZ-induced tubular injury, whereas the cytotoxic effects of STZ on pancreas islet cells were preserved. Furthermore, tubular DNA damage was evident in kidney biopsy samples from NET patients treated with STZ. Collectively, these results indicate that the mechanisms underlying the entry of STZ into cells differed between proximal tubular epithelial cells and pancreatic β-cells, which contributed to the difference in the response to STZ with the co-administration of a SGLT2 inhibitor or p53 inhibitor.

Our in vivo results revealed STZ-induced tubular injury, including the loss of transmembrane transporters, which was independent of blood glucose. The initial phenotypes of proximal tubular injury are the loss of cell polarity, cell cycle arrest, and decreases in membrane transporters, such as NaPi2a and SGLT2^10,13^. In general, when blood glucose levels become high enough to exceed reabsorptive capacity of proximal tubules, glycosuria begins to appear, typically 250–300 mg/dl of blood glucose in healthy non-diabetic controls^21^. In our NET patients, there was no evidence that blood glucose levels were elevated enough to cause the appearance of glycosuria, suggesting some abnormality in the tubular reabsorptive capacity. Furthermore, although previous findings on diabetic nephropathy were obtained using a STZ-induced type 1 diabetic model^14,15^, latent proximal tubular injury was underestimated^16^. Previous studies showed that cell cycle arrest as well as decreases in SGLT2 expression occurred after the administration of STZ^22–24^. Based on the compensatory up-regulation of SGLT2 to increase glucose reabsorption in other diabetic mouse models^23^, the down-regulation of SGLT2 after the administration of STZ indicates latent proximal tubular injury.

Our unbiased transcriptomics revealed that p53 signaling was up-regulated in STZ-treated tubular epithelial cell lines. We also found that STZ induced the phosphorylation of p53 in a dose-dependent manner in vivo and in vitro. Of note, unlike previous reports demonstrating the p53 upregulation in kidney and liver tissues in the chronic phase after STZ treatment^25,26^, p53 was found to be upregulated in the tubular epithelia in the acute phase after STZ administration. p53, a tumor suppressor protein, is a key component of the cellular response to stress, including DNA damage, oncogene expression, hypoxia, ROS, and nutrient deprivation^27^. After its phosphorylation, p53 translocates to the nucleus and transcriptionally activates a broad range of genes related to the cell cycle, apoptosis, autophagy, and metabolism^27^. We detected DNA damage within tubular epithelial cells in an STZ dose-dependent manner and showed that it persisted for 1 week after the STZ injection regardless of blood glucose levels. Previous studies identified activated p53 in tubular epithelial cells as a phenotype of STZ-induced type 1 diabetes^28^, however, STZ-induced DNA damage, not high blood glucose, may have contributed to these phenotypes.

In comparisons of DNA damage induced in vivo by cisplatin and STZ, the most prominent difference was the histological distribution of injured regions in kidney tissue. The tubular region injured by cisplatin mainly localized to the outer stripe of the outer medulla, at which the S3 segment of proximal tubules exist^29^. In contrast, the present results demonstrated that STZ-induced tubular injury was rarely detected in that region, whereas it was notably present within the outer cortex at which the S1 and S2 segments of proximal tubules exist. This supports our theory that STZ enters cells through GLUT2 and SGLT2, which are predominantly expressed in the S1/2 segment of proximal tubules.

Based on the up-regulation of p53 signaling in STZ-induced tubular injury, the pharmacological inhibition of p53 is a possible preventive strategy. Previous studies demonstrated that activated p53 signaling plays a crucial role in kidney injury in various disease models, including cisplatin nephrotoxicity and ischemia–reperfusion injury^30–36^. However, the effects of the pharmacological inhibition or genetic deletion of p53 on kidney injury are complex^36,37^. Previous studies demonstrated that the inhibition of p53 was not always beneficial, it actually exacerbated tissue injury or fibrosis^37,38^. The type of injury model and animal species may contribute to the differences observed in the response of p53 inhibition against kidney injury^37^. In STZ-induced kidney injury, we found that pifithrin-α did not mitigate DNA damage; however, it slightly improved kidney injury marker expression. Therefore, pifithrin-α did not appear to prevent STZ uptake by tubular epithelial cells, whereas subsequent p53 activation-mediated tubular injury was attenuated to some extent.

One of the main limiting factors associated with the use of anti-cancer drugs is their cytotoxicity in normal tissues, and the kidneys are the most common target organ^8^. Although serum creatinine levels did not increase in the majority of STZ-treated patients, inadequate urinary glucose excretion indicates latent proximal tubular injury. Collectively, the present results and previous findings demonstrated marked reductions in SGLT2 expression in STZ-treated mice^19,23^, which may contribute to urinary glucose excretion, while blood glucose levels did not sufficiently increase. To minimize the nephrotoxicity of anti-cancer drugs, one possible approach is to reduce the uptake of the drug into renal cells, with renal tubular-specific blockade being ideal. Consistent with previous findings, the present results showed that the pharmacological inhibition of SGLT2 partially inhibited nephrotoxicity by STZ^19^, while its cytotoxic effects on pancreatic β-cells that do not express SGLT2 were preserved. Therefore, a pretreatment with SGLT2 inhibitors has potential as a specific prophylactic approach for kidney injury.

There are several limitations to this study. First, there were discrepancies between in vivo and in vitro experimental qPCR results, particularly in the expression of transmembrane transporters in the tubular epithelia. One explanation is that loss of cell polarity and concomitant loss of expression of membrane transporters, including SGLT2, is sometimes observed in tubular epithelia under culture conditions^39,40^. Thus, the in vitro expression of these molecules may be underestimated. Second, though p53 activation is common downstream signaling of DNA damage after various insults, we could not show the p53 activation in human kidney tissue in NET patients. Lastly, while we demonstrated that dapagliflozin ameliorated STZ-induced DNA damage in tubular epithelia in vivo, its precise mechanisms especially whether STZ truly enters into the cells via SGLT2 are not directly elucidated. In addition, while STZ enters into the pancreatic β-cells via GLUT2, a bidirectional glucose transporter, the direction of GLUT2 mediated-STZ transport in the proximal tubular epithelia is unclear in our experiments, and requires further investigations in the future.

In conclusion, the present study revealed the nephrotoxicity of STZ in vitro, in vivo, and in kidney biopsy samples from NET patients. DNA damage and the subsequent activation of p53 in tubular epithelial cells are responsible for STZ-induced nephrotoxicity. SGLT2 inhibitors prevented DNA damage in tubular epithelial cells in vivo, while cell toxicity against pancreatic β-cells was preserved. This suggests that a pretreatment with a SGLT2 inhibitor is an attractive preventative option for the adverse events of STZ on kidney tissue in NET patients. Furthermore, since STZ-induced nephrotoxicity has been underestimated in experimental settings, kidney injury in STZ-induced type 1 diabetes needs to be carefully evaluated.

## Methods

### Patient samples

We retrospectively enrolled 8 NET patients treated with STZ at Kyoto Prefectural University of Medicine. All clinical characteristics were collected from medical records, including age, sex, complications, and laboratory results, and were listed in Supplementary Table 1. Human kidney samples were obtained from 2 patients by kidney biopsy. As a control, we analyzed kidney tissue from a patient with a thin basement membrane disease that primarily involved the glomerulus, not tubular tissue. Patient information was anonymized and de-identified before analyses. The entire protocol of the present study was designed in accordance with the Declaration of Helsinki. Due to its retrospective design and low risk to patients, the Ethical Committee approved the use of the following opt-out methodology. The requirement for verbal informed consent was waived and informed consent was obtained by generally accessible information as well as easy opt-out modes. The present study was approved by the Medical Ethics Committee of the University Hospital of the Kyoto Prefectural University of Medicine (Approval number: ERB-C-2169, ERB-C-2210).

### Animal experiments

Male C57BL/6 wild-type mice were purchased from Shimizu, Inc. (Kyoto, Japan). The STZ injury model was induced by an intraperitoneal injection of STZ (Cayman Chemical, MI, USA) in 0.05 M sodium citrate buffer, pH 4.5 at a concentration of 100, 150, or 200 mg/kg body weight into male mice at the age of 8–10 weeks. Mice were euthanized 1 or 7 days after the STZ injection. Littermate control mice were used for comparisons among groups. All insulin-treated groups were subcutaneously injected with insulin glargine (Wako, Osaka, Japan), and were then adjusted to the amount that maintained their fasting blood glucose at the same level (± insulin glargine 2–6 Units/kg).

To inhibit p53 in vivo, 2.2 mg/kg of pifithrin-α (ab120478, Abcam plc., Cambridge, UK) was intraperitoneally injected just before the STZ injection as previously described^41^. To inhibit SGLT2 in vivo, 1.0 mg/kg of dapagliflozin (Selleck Biotech Co., Houston USA) was dissolved and diluted with 75% normal saline (0.9% w/v NaCl) and administered by gavage 1 h before the STZ treatment. Body weights and blood glucose levels were measured at 17:00 after 8 h of fasting. Blood glucose levels were measured using a glucometer (Glutest Every, Sanwa Kagaku Kenkyusho Co., Ltd., Aichi, Japan). Mice were anesthetized with isoflurane, euthanized at the indicated time points, and blood samples were thereafter obtained from the inferior vena cava. The kidneys and pancreas were cut into samples for further analyses.

All experiments were approved by the Experimental Animals Committee of Kyoto Prefectural University of Medicine and were performed in accordance with the institutional guidelines and Guidelines for Proper Conduct of Animal Experiments by the Science Council of Japan and ARRIVE guidelines.

### Cell culture and cell viability assay

Normal rat kidney epithelial cells (NRK 52E cells) were obtained from the JCRB Cell Bank. Cells were cultured in DMEM (Wako, Osaka, Japan) containing 1% penicillin and streptomycin (Invitrogen, Carlsbad, CA) and 5% FBS (Invitrogen) at 37 °C in a humidified 5% CO_2_ and 95% air atmosphere. Cells were treated with 1, 5, 10, or 30 mM of STZ dissolved in dimethyl sulfoxide for 24 h before cell counting. The number of cells was counted using an ADAM-MC automatic cell counter (Digital Bio, Japan) that functions by using the propidium iodide staining method of dead cell staining.

### Serum blood urea nitrogen and plasma insulin measurement

Serum blood urea nitrogen (BUN) were measured using the appropriate enzymatic methods (A667-00, Serotec, Hokkaido, Japan). Insulin concentrations in the collected plasma were measured using an Ultra Sensitive Mouse Insulin ELISA Kit (Morinaga Institute of Biological Science, Inc., Kanazawa, Japan) according to the manufacturer’s instructions.

### Tissue preparation and histology

Mice were anesthetized and sacrificed, and the pancreas and kidneys were removed at the indicated time points. To prepare paraffin sections, the pancreas and kidneys were fixed with 4% paraformaldehyde and embedded in paraffin by Applied Medical Research Laboratory (Osaka, Japan). Paraffin-embedded mouse and human tissues were cut into 4-μm-thick sections. Periodic Acid-Schiff (PAS) staining was performed according to standard procedures.

### Immunohistochemistry

After deparaffinization, mouse and human paraffin sections were placed in citrate-buffered solution (pH 6.0) and boiled for 5 min to retrieve antigens. Endogenous peroxidase was quenched with 3.0% hydrogen peroxide in methanol for 20 min. Samples were blocked with 3% BSA in PBS for 30 min at room temperature and incubated with primary antibodies (Supplementary Table 2). Sections were labeled with a goat anti-rabbit HRP-conjugated secondary antibody (ab236469, Abcam). Diaminobenzidine chromogenic substrate (K3468, Agilent Technologies, Inc., Santa Clara, CA) was used for the color reaction, followed by counterstaining with hematoxylin. All sections were observed using an Eclipse E600 microscope (Nikon, Tokyo, Japan) and BZ-X700/BZ-X710 microscope (Keyence Corporation, Osaka, Japan). γH2AX-positive cells were quantified from three out of 10 consecutive non-overlapping cortical and medullary fields in each kidney under high magnification (n = 3). These three fields were randomly selected in a blinded manner.

### Immunofluorescence staining

For immunofluorescence staining, frozen sections were rehydrated and permeabilized with 0.5% Triton X-100 in PBS for 5 min. Samples were blocked with 3% BSA in PBS and sequentially incubated with the primary antibodies shown in Supplemental Table 2 overnight at 4 °C, followed by incubation with Alexa Fluor 594-conjugated secondary antibodies (Supplementary Table 2) for 1 h. Nuclear counterstaining was performed using DAPI or DRAQ5 (DR50050; BioStatus, Leicestershire, UK; 1:2000), followed by mounting in Prolong-Gold (Thermo Fisher Scientific). Images were obtained by confocal microscopy (FV1000; Olympus, Tokyo, Japan).

### RNA extraction and real‑time quantitative PCR

Total RNA was extracted from the cortex of kidneys or NRK-52E cells using TRIzol (Life Technologies, Carlsbad, CA) and Direct-zol RNA MiniPrep (Zymo Research, Irvine, CA). Two hundred nanograms of total RNA was reverse transcribed to synthesize cDNA using a PrimeScript RT reagent kit with gDNA Eraser (Takara Bio). The real-time detection of PCR products was performed using KAPA SYBR FAST qPCR Master Mix (2x) Universal (Kapa Biosystems, Wilmington, MA) and a Thermal Cycler Dice Real Time System (Takara Bio Inc.). Gene expression was quantified using β-actin as an internal control. The primers are shown in Supplementary Table 3.

### RNA sequencing

NRK52E cells, a rat proximal tubular epithelial cell line, in the STZ groups were treated with 10 mM of STZ for 24 h before RNA extraction. RNA samples were provided to the NGS core facility of the Genome Information Research Center at the Research Institute for Microbial Diseases of Osaka University for library construction and sequencing. Library preparation was performed using a TruSeq stranded mRNA sample prep kit (Illumina, San Diego, CA) according to the manufacturer’s instructions. Sequencing was performed on an Illumina HiSeq 2500 platform in a 100-bp single-end mode. Sequenced reads were mapped to the rat reference genome sequences (Rnor6) using STAR v 2.7.10a (https://github.com/alexdobin/STAR/) and uniquely mapped reads were counted by featureCounts function in the Subread package (https://subread.sourceforge.net).

### Bioinformatic analysis

Data analyses were performed using R software version 4.1.2 (https://www.R-project.org/). The edgeR package was used for a differential expression analysis^42^. Genes with FDR < 0.05 and an absolute log2 fold change > 1 were considered to be significantly differentially expressed genes. The fgsea package was used for a gene set enrichment analysis. Kyoto Encyclopedia of Genes and Genomes (KEGG) pathway analysis (https://www.genome.jp/kegg/kegg1.html) was performed using iDEP version 0.93 (http://bioinformatics.sdstate.edu/idep93/) with the generally applicable gene-set enrichment for pathway analysis (GAGE) method^43^.

### Western blot analysis

Total cell or tissue extracts were obtained using lysis buffer 17 (R&D Systems, Inc., Minneapolis, MN, USA). Proteins were denatured by heating at 95 °C for 5 min and separated by SDS-PAGE. Then, proteins were transferred onto polyvinylidene difluoride membranes (Immobilon-P IPVH00010: Millipore, MA, USA). After blocking in 5% non-fat milk or 3% BSA in TBS/0.1% Tween20 at room temperature for 1 h, the membrane was incubated with the primary antibody (Supplementary Table 2) at 4 °C overnight. After washing with TBS/0.1% Tween20, secondary peroxidase-conjugated secondary antibodies were added (7074S; Cell Signaling Technology, Boston, MA; 1:3000 at room temperature). Chemiluminescence was detected using an ECL select Western blot detection reagent (RPN2235: GE Healthcare UK Ltd, Amersham Place, England) or Clarity Max western ECL substrate (1,705,062: Bio-Rad Laboratories, Inc., Hercules, CA, USA). Signal intensities were evaluated using ImageJ software (National Institutes of Health, Bethesda, MD).

### Comet assay

The comet assay was performed using the Comet Assay Kit (Abcam ab238544) as previously described^41,44^. In brief, mouse kidneys were removed and minced in a small amount of ice-cold PBS containing 20 mM EDTA. The supernatant was passed through a 35-μm cell strainer. After centrifugation, the pellet was suspended at 1 × 10^5^ cells/ml in ice-cold PBS. Samples were mixed with comet agarose at a 1/10 ratio (v/v) and then transferred onto glass slides covered with a comet agarose base layer. After incubating with pre-chilled lysis buffer, slides were subjected to electrophoresis. Electrophoresis was performed in Alkaline Electrophoresis Solution for the alkaline comet assay and TBE Electrophoresis Solution for the neutral comet assay. After electrophoresis, slides were incubated with Vista Green DNA dye. Images were obtained by epifluorescence microscopy (IX71; Olympus, Tokyo, Japan) using the FITC filter. Ten pictures (5–15 cells per picture) were randomly taken, and the tail moment (tail length × tail % DNA/100) of 100 cells per group was calculated using Comet Score analysis software (TriTek Corp.).

### Statistical analysis

Results are expressed as the mean ± standard error (SE). Statistical analyses were performed by the unpaired *t*-test for comparisons of two variables and an analysis of variance and Dunnett’s post hoc test for comparisons of multiple variables. *P* values of < 0.05 were considered to be significant.

## Supplementary Information

Supplementary Information.

## Data Availability

RNA-seq data for all samples were deposited in the Gene Expression Omnibus under the accession number GSE215337. Secure token for reviewer access: gzanqiemjhyhdyl.
