# Supplementary material for: Streptozotocin induces renal proximal tubular injury through p53 signaling activation
Source: Sci Rep. 2023 May 29;13:8705. doi: 10.1038/s41598-023-35850-w (PMC10227064; doi:10.1038/s41598-023-35850-w)

Supplementary Information

**Streptozotocin induces renal proximal tubular injury through p53 signaling activation**

Kunihiro Nakai, Minato Umehara, Atsushi Minamida, Hiroko Yamauchi-Sawada, Yasuto Sunahara, Yayoi Matoba, Natsuko Okuno-Ozeki, Itaru Nakamura, Tomohiro Nakata, Aya Yagi-Tomita, Noriko Uehara-Watanabe, Tomoharu Ida, Noriyuki Yamashita, Michitsugu Kamezaki, Yuhei Kirita, Eiichi Konishi, Hiroaki Yasuda, Satoaki Matoba, Keiichi Tamagaki, and Tetsuro Kusaba

**3 supplementary tables, and 4 supplementary figures**

**Supplementary Table 1**. Patient characteristics

**Supplementary Table 2**. Primary and secondary antibodies for immunohistochemistry, Immunofluorescence and Western blotting

| Immunohistochemistry | Source | Catalog # | Vendor |
| --- | --- | --- | --- |
| **Primary antibody** |  |  |  |
| Megalin | Rabbit | ab76969 | Abcam |
| Phospho-histone H2A.X (Ser139) | Rabbit | 2577S | CST |
| **Secondary antibody** |  |  |  |
| Anti-rabbit antibody (HRP-conjugated) | Goat | ab236469 | Abcam |

| Immunofluorescence | Source | Catalog # | Vendor |
| --- | --- | --- | --- |
| **Primary antibody** |  |  |  |
| γH2AX | Rabbit | 2577S | CST |
| FITC-conjugated LTL |  | FL1321 | Vector Labs |
| FITC-conjugated DBA |  | FL1031 | Vector Labs |
| **Dye conjugated**  **secondary antibody** |  |  |  |
| anti-rabbit antibody | Goat | A11012 | Thermo Fisher Scientific |

| Western blotting | Source | Catalog # | Vendor |
| --- | --- | --- | --- |
| **Primary antibody** |  |  |  |
| Phospho-histone H2A.X (Ser139) | rabbit | 2577S | CST |
| Phospho-p53 (Ser15) | rabbit | 9284S | CST |
| Cleaved caspase-3 (Asp175) | rabbit | 9664S | CST |
| β-actin | mouse | A2228 | Sigma-Aldrich |
| GAPDH (HRP-conjugated) | mouse | ab105428 | Abcam |
| **Secondary antibody** |  |  |  |
| Anti-rabbit antibody (HRP-conjugated) | goat | 7074S | CST |
| Anti-mouse antibody (HRP-conjugated) | horse | 7076S | CST |

**Supplementary Table 3**. Primers for qPCR

| Mouse |  |  |
| --- | --- | --- |
| Gene | Forward | Reverse |
| *Lrp2* | AAAATGGAAACGGGGTGACTT | GGCTGCATACATTGGGTTTTCA |
| *Havcr1* | AAACCAGAGATTCCCACACG | GTCGTGGGTCTTCCTGTAGC |
| *Actb* | AGCCATGTACGTAGCCATCC | CTCTCAGCTGTGGTGGTGAA |
| *Slc34a1* | TGCCTCTGATGCTGGCTTTC | GATAGGATGGCATTGTCCTTGAA |
| *Slc5a2* | ATGGAGCAACACGTAGAGGC | ATGACCAGCAGGAAATAGGCA |

| Rat |  |  |
| --- | --- | --- |
| Gene | Forward | Reverse |
| *Lrp2* | GCAGAGATGGACAGTGAGGT | GCTGGCGAGGCTATACG |
| *Havcr1* | TGGCACTGTGACATCCTCAGA | GCAACGGACATGCCAACATA |
| *Actb* | GCGAGTACAACCTTCTTGCAG | GCCTTGCACATGCCGGA |
| *Slc34a1* | AGCTCCAGCACCTCGACATC | GCATCAGGGCCACAATGGTG |
| *Slc5a2* | GACATTCTGGTCATTGCCGC | CTGCCAAGAAGTAGCCACCA |
| *Slc2a2* | ACCAGCACATACGACACCAG | ACCATTCCGCCTACTGCAAA |


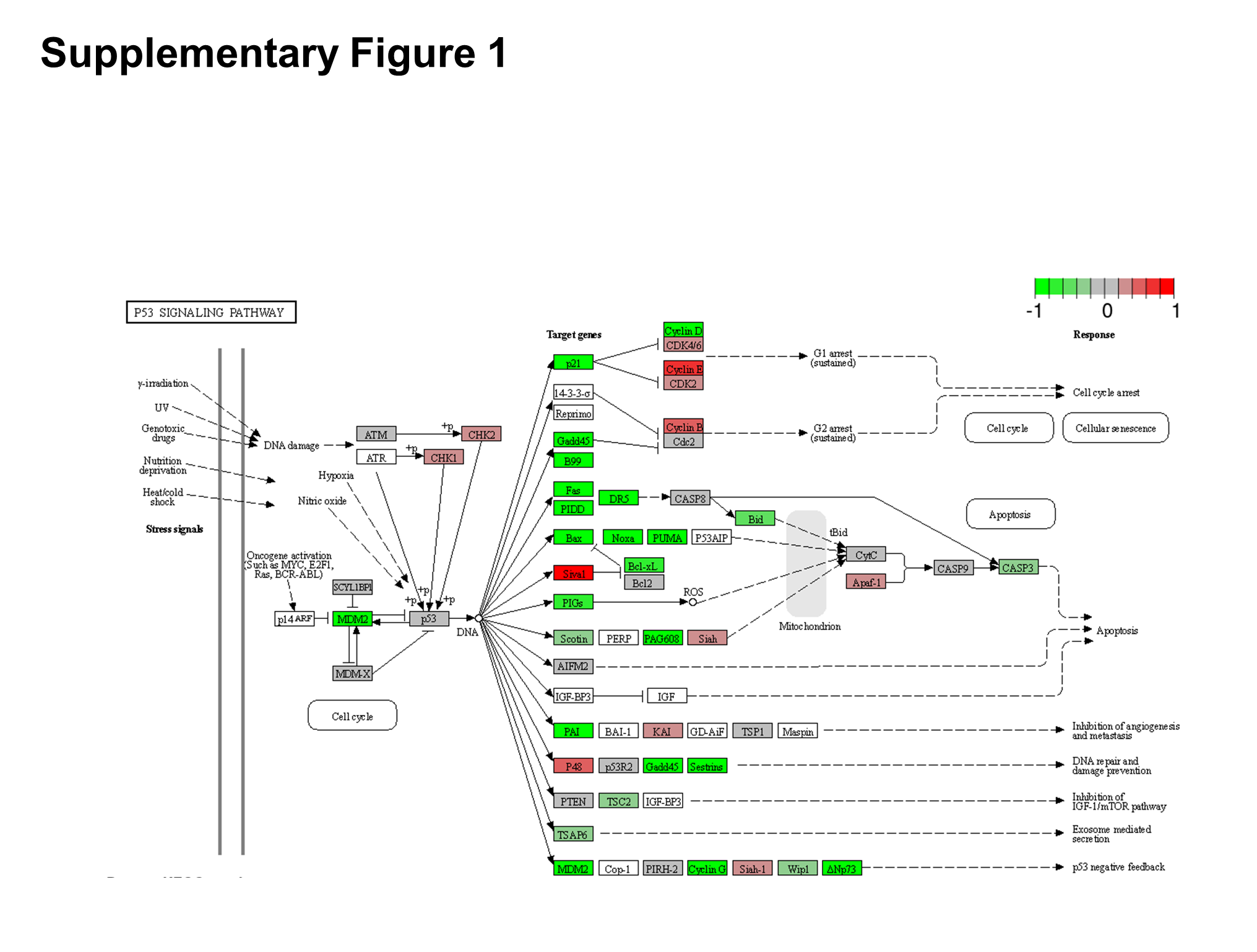
**Supplementary Figure 1. Up- or down-regulated genes in the p53 signaling pathway**


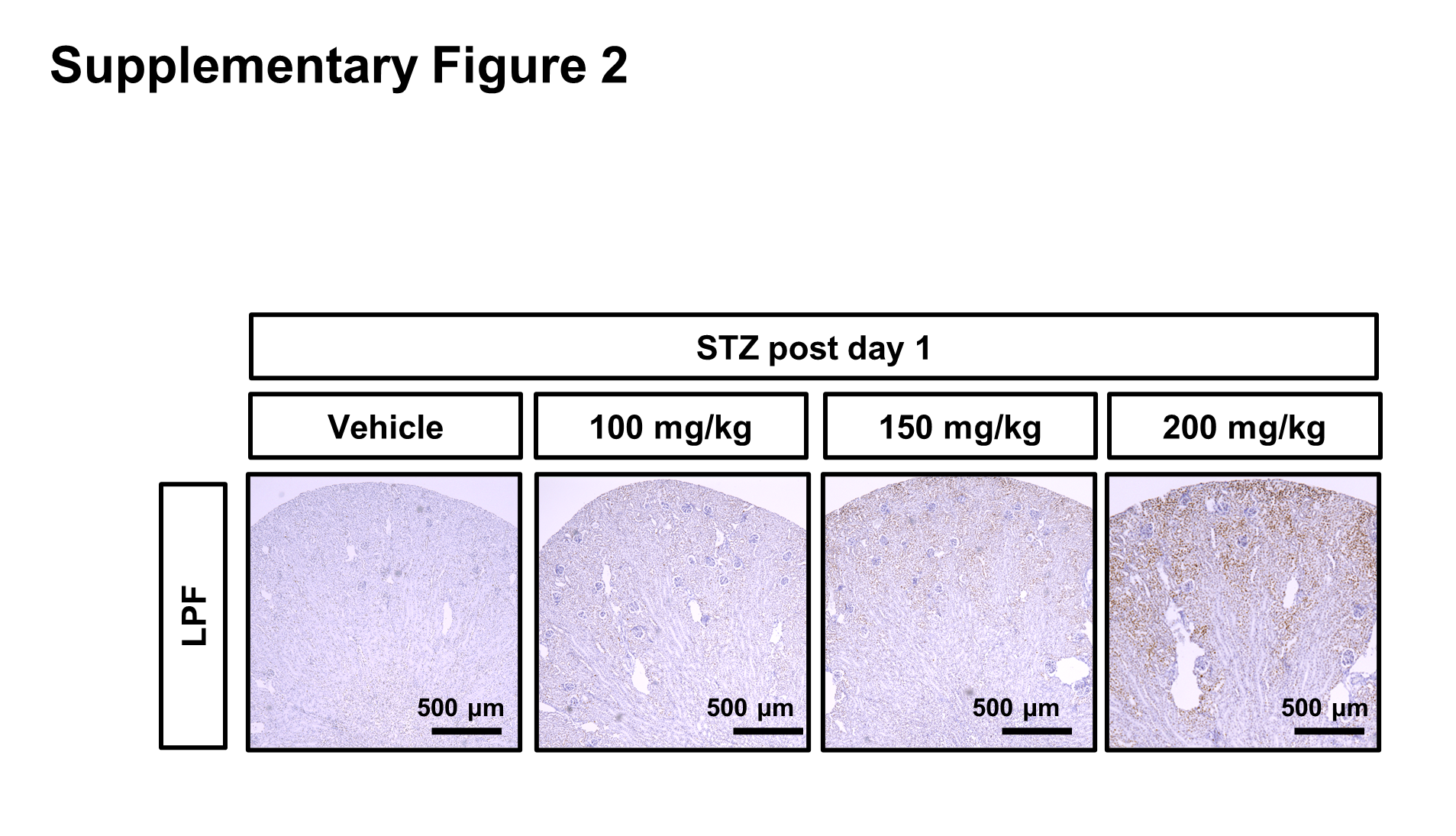
**Supplementary Figure 2. Immunostaining for γH2AX in STZ-treated mice**

Microscopic low-power field images of the kidneys of STZ-treated mice. Bar = 500 μm.


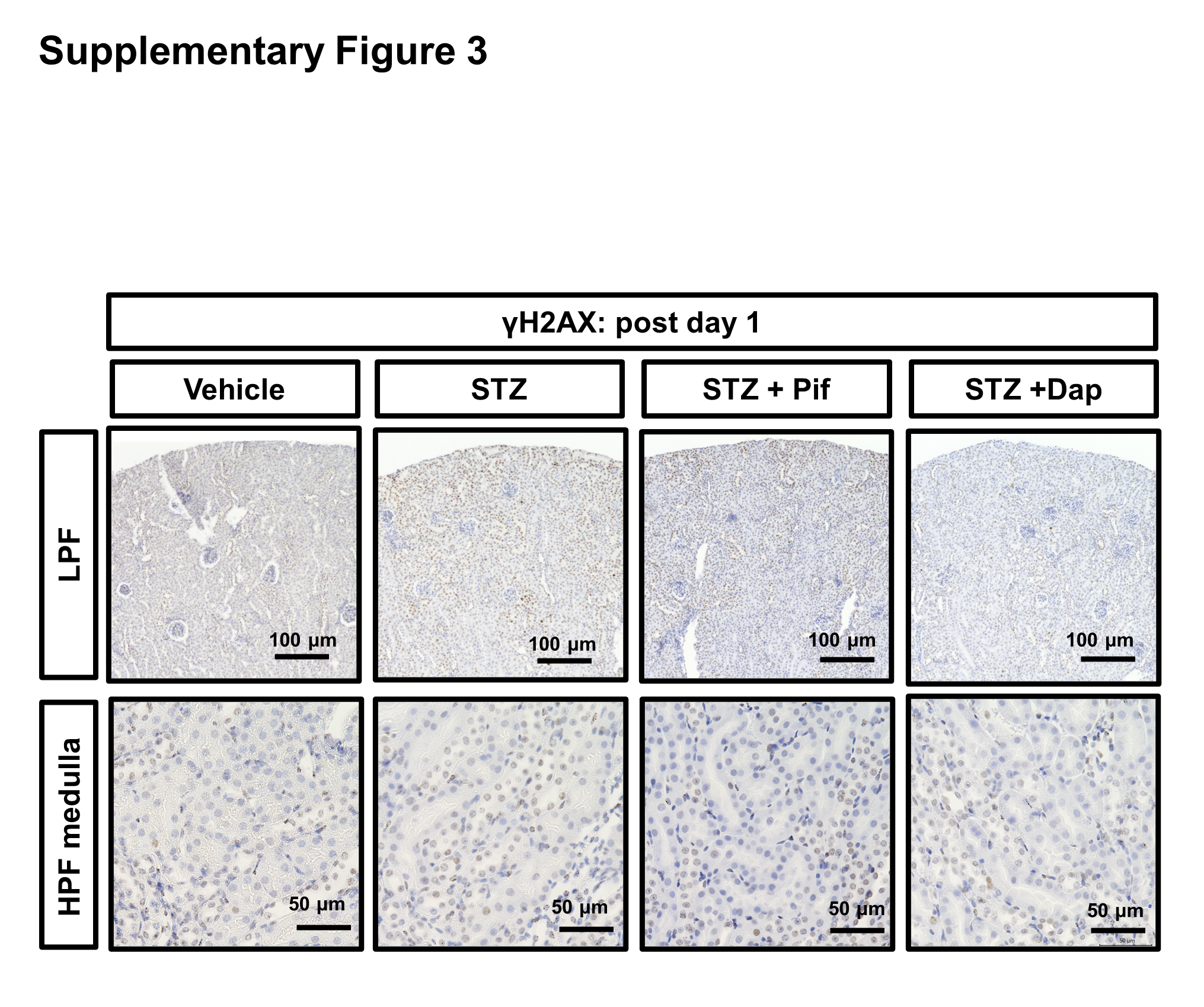
**Supplementary Figure 3. Immunostaining for γH2AX in STZ-treated mice**

(a) Microscopic low-power field images of the kidneys of STZ-treated mice. Bar = 100 μm. (b) Microscopic high-power field images of the renal medulla. Bar = 50 μm.

**Supplementary Figure 4. Unprocessed Western blots for the figures**

Figure 2e


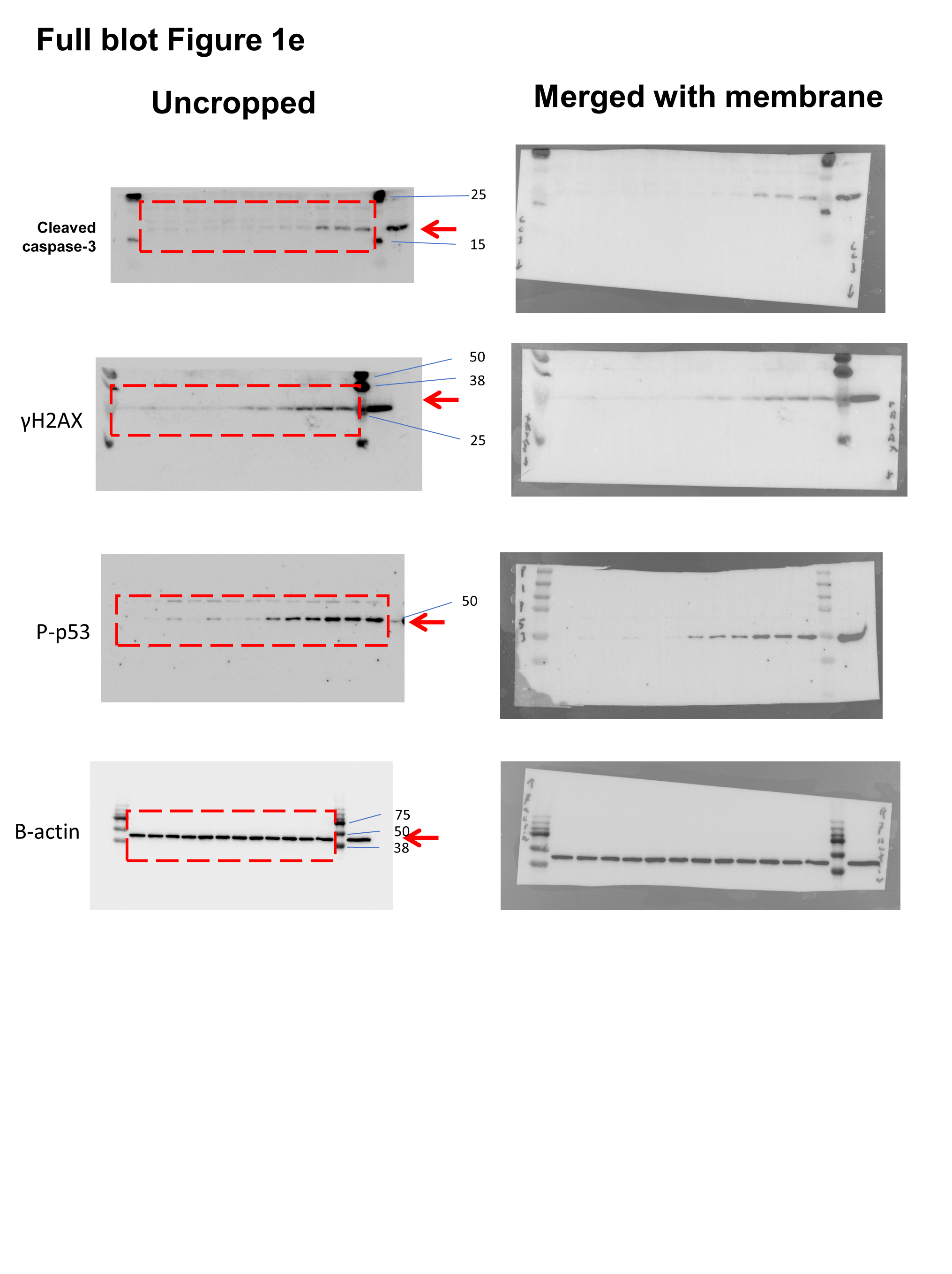


Figure 4d


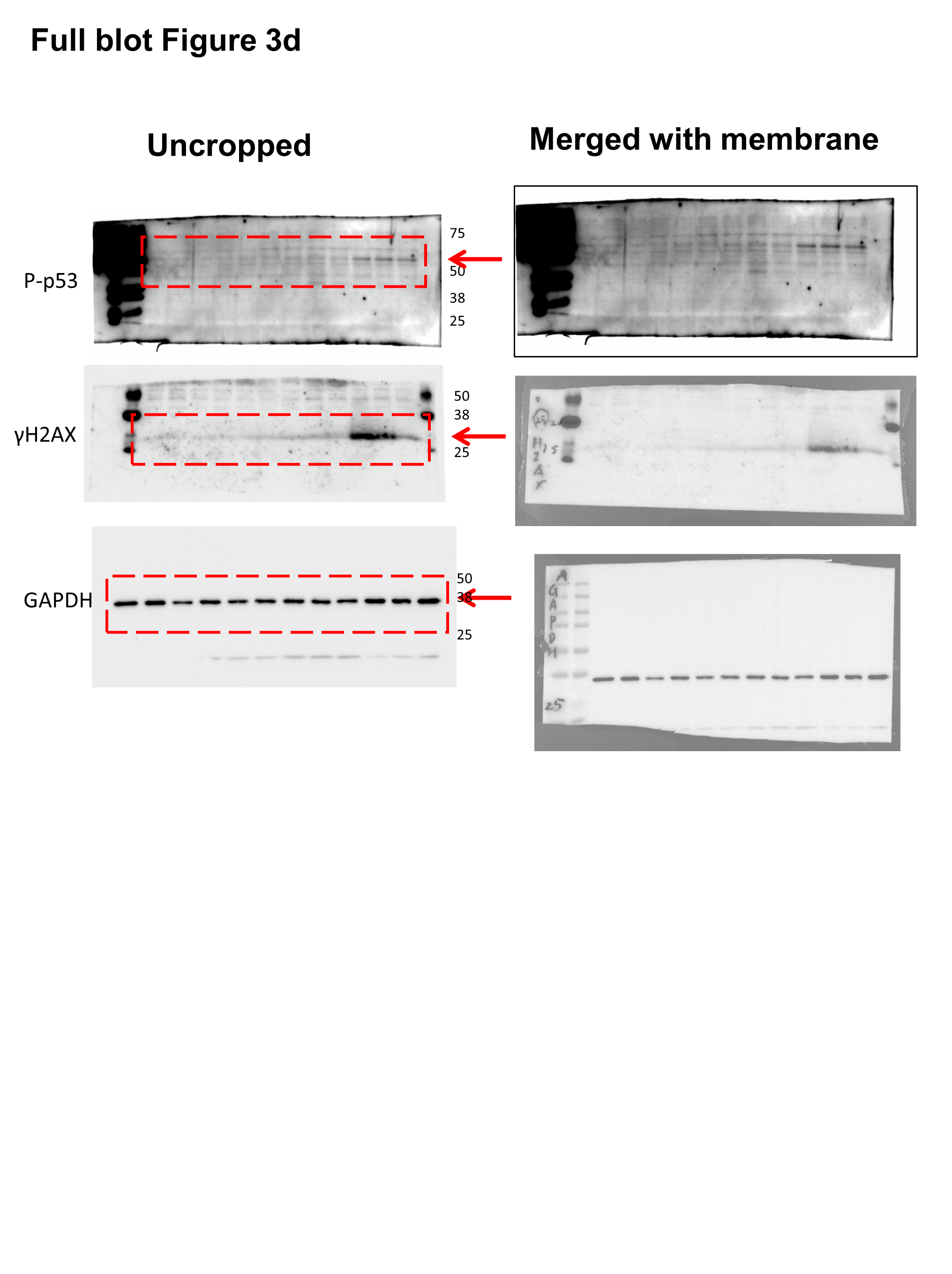


Figure 6i


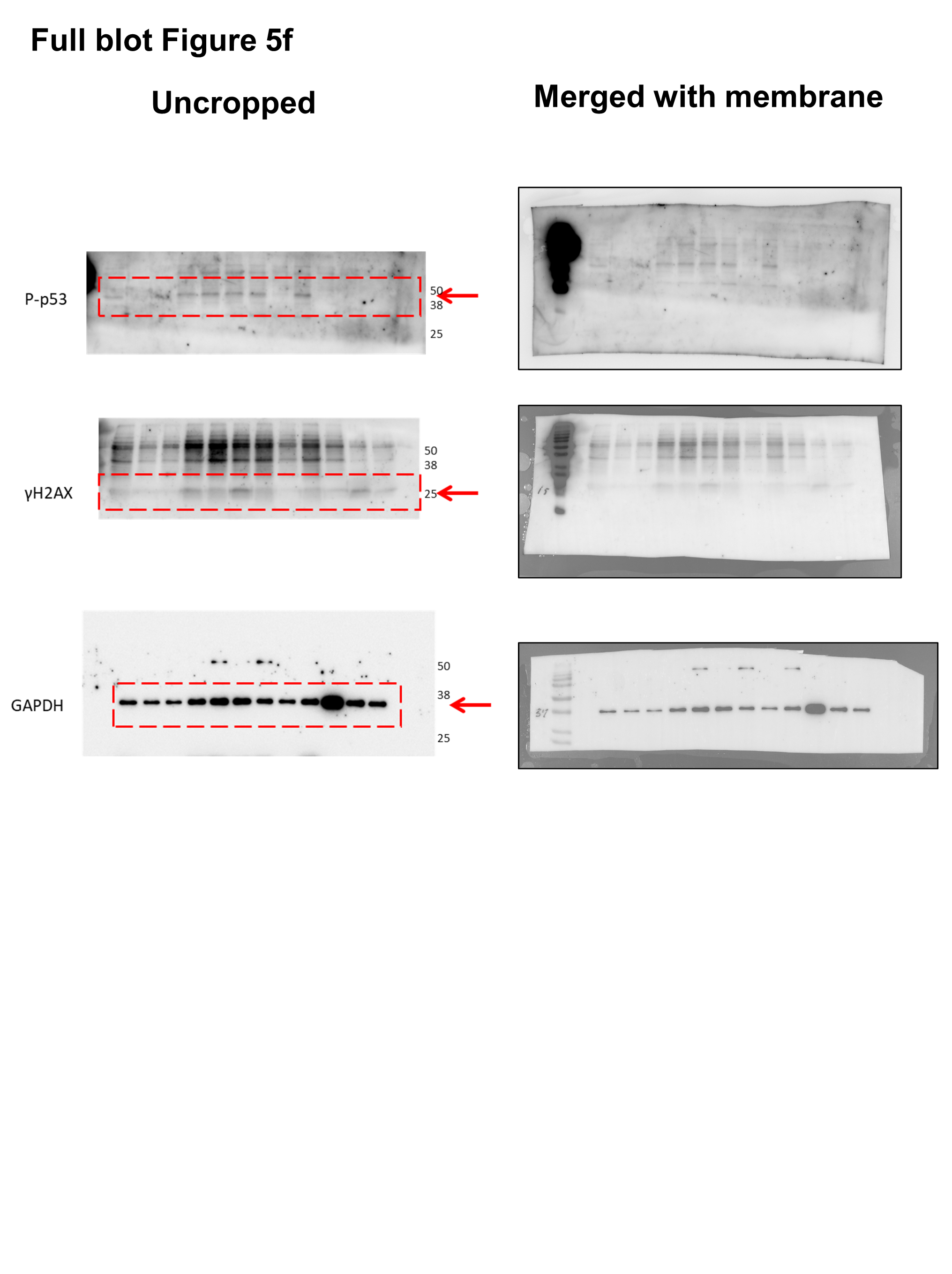

Supplement: Supplementary file 1 — Supplementary Information. [file 41598_2023_35850_MOESM1_ESM.docx]
